# Decreased Plasma Level of Cytokeratin 20 (KRT20) Is Indicative of the Emergence and Severity of Acute GvHD Irrespective to the Type of Organ Involvement

**DOI:** 10.3390/biomedicines10030519

**Published:** 2022-02-22

**Authors:** Nikolett Lupsa, Ákos Szegedi, András Gézsi, Zoltán Vuncs, Tamás Masszi, Gábor Mikala, Péter Reményi, Sara Deola, Arun Prasath Lakshmanan, Annalisa Terranegra, Edit I. Buzás, Zoltán Pós

**Affiliations:** 1 Department of Genetics, Cell and Immunobiology, Semmelweis University, 1089 Budapest, Hungary; szegak@gmail.com (Á.S.); buzas.edit@med.semmelweis-univ.hu (E.I.B.); pos.zoltan@med.semmelweis-univ.hu (Z.P.); 2 Department of Measurement and Information Systems, Budapest University of Technology and Economics, 1117 Budapest, Hungary; gezsi.andras@gmail.com; 3 Department of Hematology and Stem Cell Transplantation, South-Pest Centrum Hospital, National Institute of Hematology and Infectious Diseases, 1097 Budapest, Hungary; vuncs.zoltan@gmail.com (Z.V.); gmikala@dpckorhaz.hu (G.M.); premenyi@dpckorhaz.hu (P.R.); 4 Department of Internal Medicine and Hematology, Semmelweis University, 1088 Budapest, Hungary; masszi.tamas@med.semmelweis-univ.hu; 5 Advanced Cell Therapy Core, Sidra Medicine, Doha 122104, Qatar; sdeola1@sidra.org; 6 Precision Nutrition, Mother and Child Health Department, Sidra Medicine, Doha 122104, Qatar; alakshmanan@sidra.org (A.P.L.); aterranegra@sidra.org (A.T.); 7 Immunproteogenomics Extracellular Vesicle Research Group of the Hungarian Academy of Sciences-Semmelweis University, 1089 Budapest, Hungary; 8 Extracellular Vesicle Research Group, Hungarian Center of Excellence Molecular Medicine, 1089 Budapest, Hungary

**Keywords:** acute graft versus host disease, aGvHD, biomarker, cytokeratin 20, KRT20, K20

## Abstract

Accurate risk prediction of acute graft versus host disease (aGvHD) is currently an unmet clinical need. This study sought to analyze whether three plasma proteins expressed in a largely skin- and gut-restricted manner would be affected by the development of acute cutaneous and gastrointestinal aGvHD. The diagnostic sensitivity, specificity, and prognostic value of plasma cytokeratin-15 (KRT15) cytokeratin-20 (KRT20), and occludin (OCLN) were evaluated in a discovery and a validation cohort using ELISA in comparison with elafin (PI3) and regenerating family member 3 alpha (REG3A), two established markers of skin- and gut aGvHD. The discovery cohort (*n* = 39) revealed that at the time of diagnosis, plasma KRT20 showed a progressive decrease from unaffected individuals to patients with single-, and patients with multi-organ aGvHD. KRT20 was affected by cutaneous (*p* = 0.0263) and gastrointestinal aGvHD (*p* = 0.0242) independently and in an additive manner. Sensitivity and specificity of KRT20 for aGvHD involving both target organs (AUC = 0.852) were comparable to that of PI3 for skin-aGvHD (AUC = 0.708) or that of REG3A for gut-aGvHD (AUC = 0.855). Patient follow-up in the validation cohort (*n* = 67) corroborated these observations (*p* < 0.001), and linked low KRT20 to grade 2+ disease (*p* < 0.001), but failed to confirm low KRT20 as an independent risk factor. These data established a link between low plasma KRT20 levels and moderate to severe aGvHD involving multiple target organs.

## 1. Introduction

Despite significant advances in prophylaxis and therapy, acute graft versus host disease (aGvHD) remains one of the most common and life-threatening complications of allogeneic hematopoietic stem cell transplantation (aHSCT) [1]. Acute GvHD develops on the grounds of major and minor allele histocompatibility mismatches between the allogenic donor and recipient, leading to immune-mediated, extensive tissue damage which affects a select set of target organs (most frequently the skin and the gastrointestinal tract of the host) [1]. There is consensus that accurate risk prediction and early diagnosis of aGvHD could significantly improve patient care.

Current approaches of aGvHD risk assessment and prophylaxis are poorly standardized across countries and centers [2]. Therefore, the need for establishing consensus guidelines regarding optimal prevention strategies has emerged [3]. Unfortunately, most current recommendations depend on clinical factors that are insufficient or difficult to use for precise risk stratification, such as the applied conditioning regimen, donor-recipient relationship, and HLA match status. Thus, accurate risk prediction is an unmet clinical need [3]. Diagnosis of aGvHD, and its gastrointestinal, cutaneous, or other organ manifestations, is based on simple diagnostic criteria such as the demonstration of the characteristic maculopapular skin rash, assessment of the extent of diarrhea, histology performed on organ biopsies, or routine lab tests analyzing liver function. Nevertheless, as our understanding of the events leading to the development of aGvHD has been deepened significantly, in recent years, a large number of alternative markers has been proposed (although most of them failed to establish themselves in clinical practice).

In recent years, several studies have convincingly demonstrated that key initiator of aGvHD is gut barrier damage caused by the cytotoxic conditioning regimen introducing aHSCT. Barrier damage is rapidly exploited by various members of the gut microbiota, leading to an exacerbated inflammatory response, cytokine storm, and subsequent activation of mismatch antigen-specific T cells and other leukocytes derived from the allogeneic graft. Based on this information, over the last decades, considerable research efforts have been devoted to identify and validate novel and reliable molecular biomarkers for aGvHD diagnosis, prognosis, risk assessment, and prediction of therapy response [4]. A wide range of approaches have been utilized for the analysis of graft composition, inflammation- or T-cell response-related gene polymorphisms (e.g., SNPs in the genes encoding IL-6 and IFNγ) [5,6], plasma proteins (e.g., ST2, REG3A, TNFR1) [7,8,9], microRNAs (e.g., miRNA-155) [10], extracellular vesicles [11], or microbial DNA sequences [12,13] as predictors or indicators of several aspects of aGvHD, such as the characteristic reorganization of the gut microbiota, gut barrier damage, cytokine storm, or graft leukocyte activation (reviewed in [14,15]). Many of these approaches provided proof of principle or showed considerable promise, while the clinical applicability of others turned out to be a mixed bag, as their establishment is often hampered by issues related to method standardization, lack of certain analytic instruments or technical know-how in clinical centers, or limited cost-efficiency.

Currently, circulating plasma proteins represent arguably the most versatile aGvHD biomarkers, as blood samples are routinely taken. Hence, these markers are readily available for analysis. Some of them have been proven as accurate prognostic and diagnostic markers in multicenter studies, and straightforward prognostic algorithms could be developed based on these assays [7,16]. Among such aGvHD markers, several are linked to the affected target organs. First among them is arguably regenerating islet-derived protein 3A (REG3A), a well-characterized, GI-tract-restricted antimicrobial protein, expressed predominantly by Paneth cells and well-known as an established biomarker covering the whole course of aGvHD. Several studies have focused on analyzing the clinical value of REG3A in aGvHD, and it has been successfully validated as a biomarker of lower gastrointestinal aGvHD and a predictor of long-term non-relapse mortality [17,18]. In addition to REG3A, circulating elafin (peptidase inhibitor 3, hereafter PI3) is another tissue-restricted plasma biomarker of aGvHD, produced by keratinocytes in highly inflamed epidermis of the skin and released in the blood stream upon TNFα and IL-1 release. It has been demonstrated that elevated plasma level of PI3 correlates with cutaneous aGvHD and disease severity [19], and these initial findings have been confirmed by others as well [20]. Nevertheless, PI3 is inferior to REG3A as a prognostic marker when determining non-relapse mortality or overall survival [21], and it has been suggested that PI3 may be inappropriate for the differential diagnosis of skin damage induced by cutaneous aGvHD [22]. Finally, the intestinal-type fatty acid-binding protein (FABP2) is another gut-restricted antimicrobial protein the dysregulation of which has been also reported in the blood in aGvHD [23,24]. Taken together, there is convincing evidence that expression and/or release of tissue-restricted proteins, derived from aGvHD target organs, present in the blood circulation, may be altered by aGvHD, and this may be exploited in the risk assessment and diagnosis.

Following this thread, in this study we sought to identify novel plasma aGvHD biomarkers possibly affected by aGvHD and its two most frequent organ manifestations, namely cutaneous and gastrointestinal aGvHD. We selected the skin- and gut-restricted proteins cytokeratin 20 (KRT20), occludin (OCLN), and cytokeratin 15 (KRT15) and evaluated their diagnostic and prognostic value in comparison with REG3A, PI3, and FABP2 as established and emerging organ-restricted aGvHD markers. Here, we report the identification and initial validation a low plasma cytokeratin 20 (KRT20) level as a novel biomarker of aGvHD.

## 2. Materials and Methods

### 2.1. Patients

Adult patients receiving aHSCT for various indications were recruited for the study at the Department of Hematology and Stem Cell Transplantation, South Pest Central Hospital—National Institute for Hematology and Infectious Diseases, Budapest, Hungary. Patient recruitment, aHSCT, and blood sample collection were carried out after receiving informed consent, following an IRB-approved study protocol, according to the principles of the Declaration of Helsinki. All patients were registered in the European Bone Marrow Transplantation (EBMT) Registry. Patient characteristics are summarized in Table 1 and Table 2. Please refer to Supplementary Tables S1 and S2 for detailed patients’ data including age, gender, therapy indication, donor-recipient relatedness, MHC mismatch, conditioning regimen, GvHD prophylaxis given, acute GvHD developed, GvHD grade, and organ involvement.

### 2.2. Study Design

**Figure 1:**
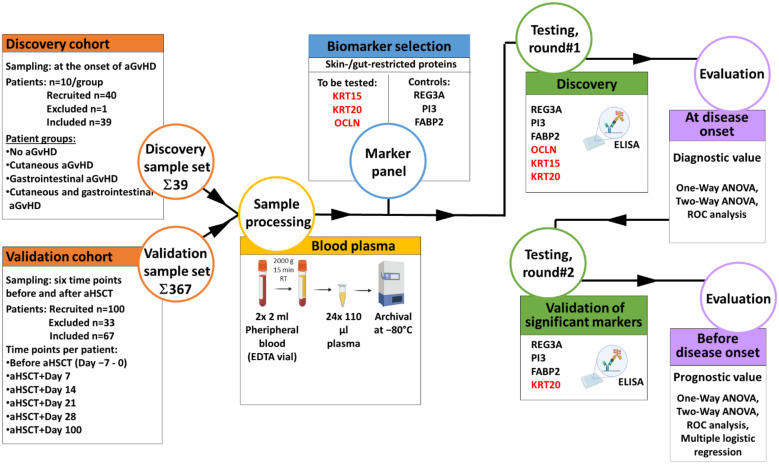
Overview of the study design. Brief overview of the study design summarizing the composition and relationship of the discovery and validation sets, the parameters of sample collection and processing, the markers studied, the reference markers included, and the statistics applied.

A discovery and validation cohort study was conducted by assigning aHSCT patients to two distinct, non-overlapping patient cohorts. The discovery cohort was evaluated in a retrospective case-control study to identify novel diagnostic markers of GvHD (i.e., plasma proteins affected by the onset of aGvHD and its distinct organ manifestations, such as cutaneous and gastrointestinal aGvHD (Figure 1)). This cohort consisted of 40 patients belonging to four age- and gender-balanced groups depending on organ involvement (i.e., (i) aHSCT patients developing no aGvHD, (ii) cutaneous aGvHD, (iii) gastrointestinal aGvHD, or (iv) both (*n* = 10 each)). Out of 40 patients recruited, 1 patient was excluded due to inadequate sample quality, leaving 39 patients included in the study. A second, independent validation cohort was also recruited, and analysed in a prospective cohort study to validate the diagnostic value of aGvHD markers identified in the discovery sample set. To investigate their prognostic value, the latter was assessed at multiple time points before disease onset (Figure 1). The validation cohort consisted of 100 patients, out of which 67 were included into the second analysis as they met eligibility criteria by providing one pre-transplant and at least three post-transplant blood samples for the validation sample set.

### 2.3. Sample Collection

For the discovery sample set, blood samples were taken at the time of aGvHD diagnosis (cutaneous aGvHD, GI aGvHD, or both cutaneous and GI aGvHD), or around day 25 post aHSCT (control group developing no aGvHD), one sample per patient, for a total of 39 samples. For the validation sample set, blood was collected once on the week before aHSCT (day -7-0 before aHSCT), and in five subsequent time points (days 7, 14, 21, 28, and 100 post aHSCT), four to six samples per patient, 367 samples total (Figure 1). Peripheral blood was collected by venipuncture using VACUETTE^®^ Acid Citrate Dextrose (ACD-A) tubes (Greiner Bio-One, Kremsmünster, Austria). Samples were transported and stored at room temperature (RT) for not more than 4 h before further processing. Samples were centrifuged 2000× *g* for 15 min at RT, the plasma fraction was collected, split into 110 uL aliquots, and stored at −80 °C until further use.

### 2.4. ELISA

Frozen plasma aliquots were thawed for ELISA at RT, used once only, with no repeated freeze-thaws allowed, and evaluated immediately. Plasma protein concentration was determined using Human FABP2/I-FABP DuoSet ELISA (for FABP2), Human Trappin-2/Elafin DuoSet ELISA (for PI3), Human Reg3A DuoSet ELISA Reg3a (for REG3A, all from R&D, Minneapolis, MN, USA), Human Occludin (for OCLN), and KRT15/CK15/Cytokeratin 15 ELISA Kit (for KRT15, both from LSBio, Seattle, WA, USA), and a Human Cytokeratin 20 ELISA Kit (for KRT20, obtained from Novus, Littleton, CO, USA). All ELISA tests were executed according to manufacturer’s instructions. Optimal plasma dilutions were pre-determined in pilot experiments. Plasma samples were diluted for distinct ELISA assays, as follows: Reg3α, both GI and cutaneous aGVHD manifestation group 1:1000, other samples 1:30, PI3 1:100, FABP2 1:1, KRT15 1:300, OCLN 1:10 and KRT20 1:1.

### 2.5. Statistical Analysis

Data analysis was carried out with GraphPad v9.11, as indicated. ANOVAs/*t*-tests were applied to compare plasma protein levels between patient groups. Data sets with non-normal distribution were log-transformed to achieve normality, or analyzed with their respective non-parametric assays. Marker sensitivity and specificity were determined by receiver operating characteristic (ROC) analysis. Multiple logistic regression was applied to test the influence of individual clinical parameters on the plasma protein levels of GvHD markers. *p* values smaller than 0.05 were considered statistically significant.

## 3. Results

### 3.1. Marker Selection

We selected six markers for the study based on literature data and data mining of the Protein Atlas public gene expression database [25]. We assumed that markers displaying a largely tissue-restricted expression (i.e., markers expressed predominantly by the skin and/or the gut, also known to be actively secreted in the extracellular space and/or released upon tissue damage, and known to have detectable levels in the blood plasma) may be subject of changes due to the rise of GvHD in these target organs, and hence may be considered as promising targets for biomarker discovery in the context of acute GvHD.

First, we selected the largely skin- and gut-restricted protein cytokeratin 15 (thereafter termed as KRT15) to be tested as a novel marker candidate for cutaneous and/or gastrointestinal aGvHD, as KR15-expressing basal epithelial cells were suggested to be selectively targeted in experimental cutaneous aGvHD [26]. However, it is unknown if this translates to the human setting or changes in the circulation. Second, we chose the gut-restricted protein occludin (hereafter OCLN) to be analyzed in GI-aGvHD, considering the pivotal role played by occludin in the maintenance of tight junctions and epithelial barrier function and the available murine data suggesting that down-regulation of occludin [27] occurs in GI-aGvHD models. As intestinal barrier dysfunction is an essential, introductory step in the development of aGvHD, and there are no human data available on circulating OCLN proteins, we set out the clarify this issue. Finally, cytokeratin 20 (hereafter KRT20) was also selected for investigation, considering the data suggesting that this protein is mostly confined to differentiated luminal gut epithelial cells [28,29] deeply involved in the maintenance of the barrier, however, human clinical data on KRT20 levels in aHSCT patients are also lacking.

Finally, three additional proteins were also included in the study, serving as reference during the assessment of diagnostic value, sensitivity, and specificity of KRT15, OCLN, and KRT20. These three controls consisted of regenerating family member 3 alpha (REG3A), an established marker of GI-aGvHD, protease inhibitor 3 (PI3, also known as elafin, or trappin-2), described as plasma protein marker associated with the development of cutaneous aGvHD, and fatty-acid binding protein 2 (also known as intestinal-type fatty acid-binding protein (FABP2)) an emerging marker of aGvHD with gut-restricted expression. All markers were analyzed in a discovery and validation cohort study, as shown in Figure 1.

### 3.2. REG3A and PI3 Validate Patient Selection and Study Design

**Figure 2:**
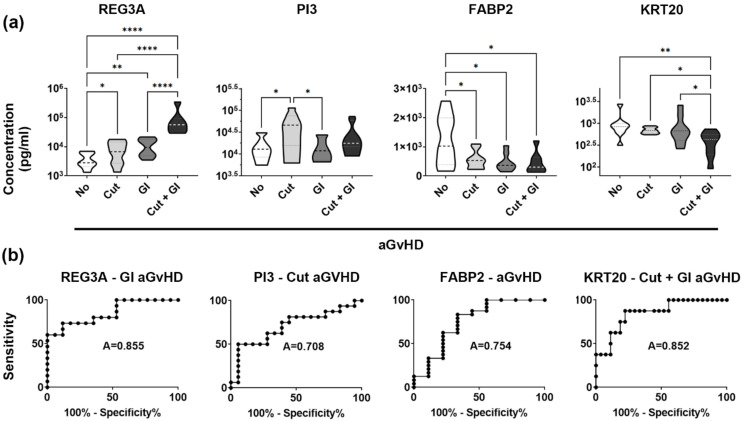
Plasma levels, sensitivity, and specificity of established and emerging tissue-restricted aGvHD biomarkers in comparison with KRT20. (**a**) REG3A, PI3, FABP2, and KRT20 protein concentration in the blood plasma of four aHSCT patient groups (*n* = 10 each) affected by distinct organ manifestations of aGvHD. Abbreviations: No, no aGvHD developed; Cut, cutaneous; GI, gastrointestinal aGvHD. Protein levels were determined at the time of aGvHD diagnosis, using commercial ELISA assays (One-Way ANOVA, stars indicate results of post hoc tests; * *p* < 0.05, ** *p* < 0.01, **** *p* < 0.0001). (**b**) Sensitivity and specificity of the same four markers linked to the respective organ manifestations of GvHD (receiver operating characteristic, values indicate area under the curve).

In line with literature data, analysis of the discovery set confirmed that at the time of aGvHD diagnosis, development of gastrointestinal aGvHD associated with markedly elevated levels of REG3A (One-Way ANOVA *p* < 0.0001) in the blood stream (Figure 2a). Similarly, development of cutaneous aGvHD was linked to increased plasma PI3/elafin levels (One-Way ANOVA *p* = 0.0125, Figure 2a). As for FABP2, a uniform downregulation (One-Way ANOVA *p* = 0.0160) was apparent in all analysed GvHD patient groups regardless of organ involvement compared to patients unaffected by aGvHD (Figure 2a). Taken together, these data confirmed that the discovery set was representative of patient cohorts analysed in others’ similar studies¸ establishing that tissue-restricted aGvHD markers behaved as expected.

### 3.3. Low Plasma KRT20 Levels in aGvHD with Skin and Gut Involvement

In contrast, analysis of plasma OCLN (One-Way ANOVA *p* = 0.2309) and KRT15 (One-Way ANOVA *p* = 0.1754) levels, two proteins linked to the gut and the skin, respectively, did not reveal any obvious trends or significant associations between these proteins and aGvHD, or any of its particular organ manifestations (not shown). However, plasma KRT20, a gut-restricted protein that has not yet been linked to aGvHD, was obviously affected (One-Way ANOVA *p* = 0.0158) by the disease (Figure 2a). Mean KRT20 levels showed a steady decrease from unaffected patients to cases of cutaneous and gastrointestinal GvHD, with patients displaying both skin and gut involvement being the most strikingly affected, exhibiting significantly less plasma KRT20 than any other patient groups analyzed (see Figure 2a). We next tested if KRT20, being a plasma protein with largely gut-restricted expression, would show a connection to gastrointestinal GvHD as an independent event, occurring both in the presence or absence of cutaneous manifestations. The experimental design allowed for testing GI and skin aGvHD as independent events regardless of the clinical emergence of the other, as the discovery set consisted of four patient groups covering all possible combinations of tissue involvements. In line with literature data, REG3A associated with GI GvHD independently of cutaneous GvHD (Two-Way ANOVA *p* < 0.0001), and so did PI3 with cutaneous aGvHD independently of gut manifestations (Two-Way ANOVA *p* = 0.0058). In contrast, such an analysis revealed that KRT20 levels were lower in both cutaneous (Two-Way ANOVA *p* = 0.0263) and gastrointestinal aGvHD (Two-Way ANOVA *p* = 0.0242) occurring as independent events. There was no evidence of any interaction between these two events (Two-Way ANOVA *p* = 0.2050), however. This suggests that plasma KRT20 is lowered by both organ manifestations independently, in an additive, but not synergistic, manner.

### 3.4. KRT20 Is a Sensitive and Specific Marker of aGvHD Affecting Multiple Tissues

We next tested the sensitivity and specificity of these proteins as markers for the organ manifestations seemingly linked to them. ROC analysis disclosed that in the discovery set, sensitivity and specificity of REG3A (AUC = 0.8549) as a marker of GI involvement, that of PI3 as a marker linked to cutaneous aGvHD (AUC = 0.7083), and that of FABP2 associated with aGvHD in general (AUC = 0.7546) were comparable to that of KRT20 (AUC = 0.8519) as a marker linked to cases with aGvHD affecting both skin and the gut (Figure 2b). Taken together, low plasma KRT20 concentration associated clearly with aGvHD involving multiple target organs.

**Figure 3:**
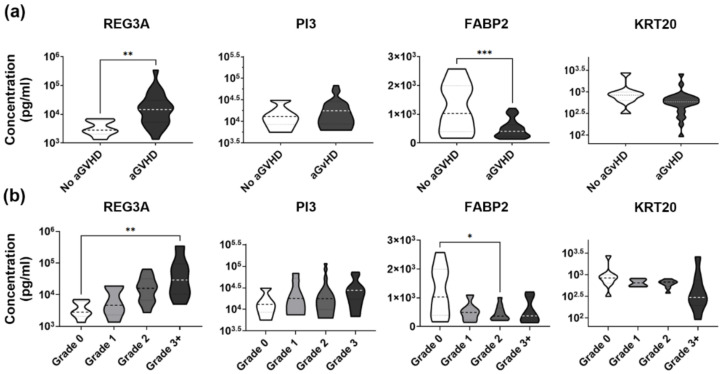
Plasma levels of select plasma proteins under the influence of aGvHD development and disease severity, as measured at the time of diagnosis, by ELISA assays. (**a**) Concentration of four plasma proteins depending on the presence and absence of aGvHD (*t*-tests, stars indicate significance; ** *p* < 0.01, *** *p* < 0.001). (**b**) Relationship between plasma protein concentration and GvHD severity, as measured by grade (One-Way ANOVA, stars indicate results of post hoc tests; * *p* < 0.05, ** *p* < 0.01).

Finally, we also tested if, in the discovery set, the analysed markers would relate to the development of aGVHD as such (i.e., regardless of the extent and type of organ damage), or if they would be affected by aGVHD depending on grade. We found that at the time of aGvHD diagnosis, decreased FABP2 was most affected by the development of aGVHD (Figure 3a, *t*-test *p* = 0.0009), while REG3A was the marker most influenced by grade (Figure 3b One-Way ANOVA *p* = 0.0010). In contrast, at least in the discovery set, we did not find robust evidence linking low plasma KRT20 to all cases of aGvHD regardless of the organs involved (Figure 3a, *t*-test *p* = 0.0905), or to high-grade aGvHD (Figure 3b, Kruskal-Wallis One-Way ANOVA *p* = 0.0626).

### 3.5. Validation Set Confirms Robust Association between Low Plasma KRT20 and Widespread Organ Damage in aGvHD

We next analysed whether these findings, implying the existence of a link between low plasma KRT20 and the rise of severe cutaneous and gastrointestinal GvHD, could be validated in a larger, independent patient cohort. We also wanted to clarify if they held true in time points other than at the time of GvHD diagnosis (i.e., when re-analysed in sample set collected at six time points before (days -7-0) and after (days 7, 14, 21, 28, and 100) aHSCT).

**Figure 4:**
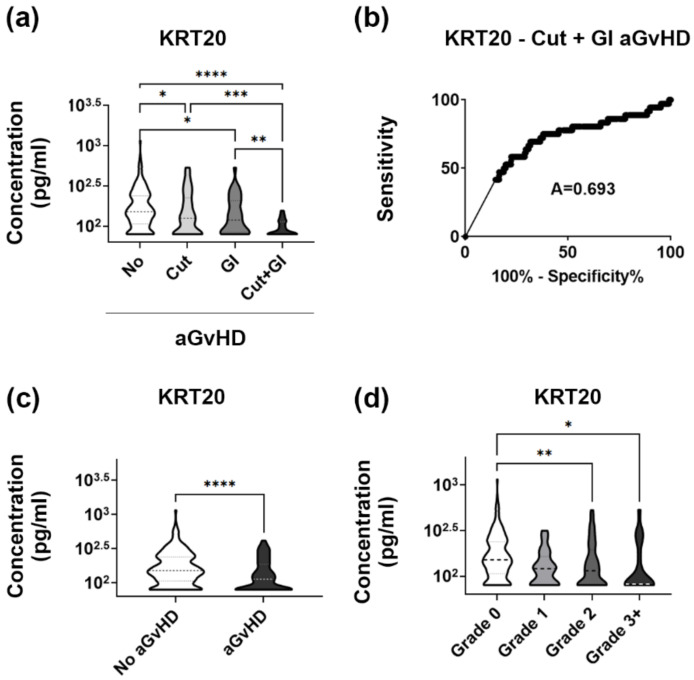
Validation of KRT20 as a putative biomarker of aGvHD with multiple organ involvement in a validation cohort. (**a**) KRT20 levels in aHSCT patients without GvHD compared to patients developing aGvHD affecting the skin, the gut, or both the skin and the gut in the validation set. Summarized results of a cohort study analysing blood protein levels at select time points before and after aGvHD diagnosis, using commercial ELISA assays (Kruskal-Wallis test, stars indicate results of post hoc tests; * *p* < 0.05, ** *p* < 0.01, *** *p* < 0.001, **** *p* < 0.0001) Abbreviations: No, no aGvHD; Cut, cutaneous aGvHD; GI, gastrointestinal aGvHD. (**b**) Sensitivity and specificity KRT20 as a marker of aGvHD involving the skin and the gut (receiver operating characteristic, area under the curve shown). (**c**) Plasma KRT20 in aHSCT patients affected or unaffected by aGvHD (*t*-test; **** *p* < 0.0001). (**d**) KRT20 levels depending on aGvHD grade (One-Way ANOVA, stars indicate results of post hoc tests; * *p* < 0.05, ** *p* < 0.01).

Analysis of the validation set confirmed the link between low KRT20 and widespread organ damage, as observed in the discovery set. KRT20 levels decreased (Mann-Whitney *p* = 0.0002) from unaffected aHSCT patients to GvHD involving multiple target organs (Figure 4a). The same applied to sensitivity, and specificity of KRT20 as a marker of such cases (Figure 4b). The existence of a strong link between decreased KRT20 and gastrointestinal involvement as an independent event (Two-Way ANOVA *p* = 0.0089) was confirmed, by the validation cohort, as well. In contrast, inclusion of a large array of blood samples obtained both before and after aGvHD diagnosis obviously decreased the influence of cutaneous GvHD (Two-Way ANOVA *p* = 0.0891) on plasma KRT20. This implies that this latter factor modifies KRT20 in a time-dependent manner, being less pertinent in time points other than at diagnosis.

### 3.6. Validation Set Discloses Lowered Plasma Levels of KRT20 in Medium to High Grade aGvHD

Finally, the validation set revealed similar patterns of KRT20 expression when comparing unaffected vs. GvHD patients, and patients grouped according to disease severity, as well. In the discovery set, concentration of plasma KRT20 was found to be significantly lower in aGvHD patients’ blood samples than in those obtained from unaffected individuals undergoing aHSCT (*t*-test *p* < 0.0001, Figure 4c). In line with this, gradual reduction of KRT20 from GvHD grade 0 to grade 4 observed in the discovery set was reproduced, as well reaching statistical significance (Kruskal-Wallis ANOVA *p* < 0.0001, Figure 4c). Considering that the sample size in the validation set was considerably larger than in the discovery set (367 vs. 40), increased robustness between apparent differences may not be surprising. On the other hand, it must be noted that sampling has not been incorporated in these evaluations as a factor.

### 3.7. Analysis of the Kinetics of KRT20 Dysregulation in Severe aGvHD: Possible Prognostic Value?

Finally, we tested if plasma KRT20 was different in blood samples collected at different time points pre- and post-aHSCT, depending on organ involvement, before and after diagnosis of GvHD. As shown in Figure 3a, aHSCT patients developing no GvHD and patients affected by a combination of skin and gut GvHD displayed the largest difference in terms of circulating KRT20 overall.

**Figure 5:**
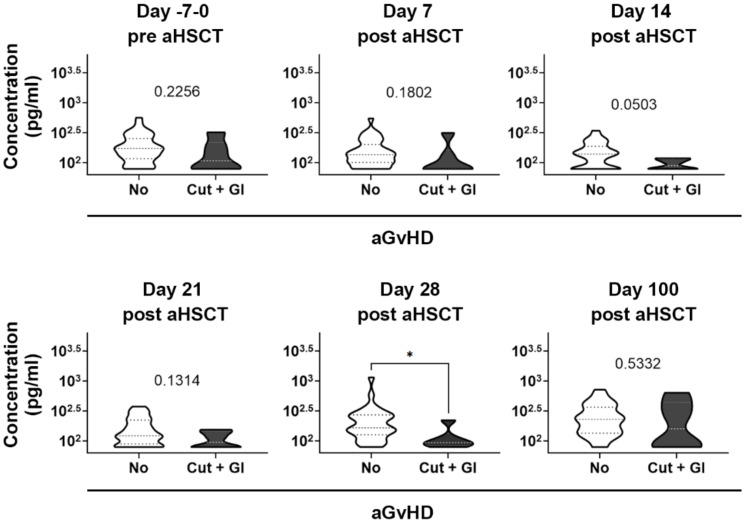
Follow-up analysis of the kinetic changes of plasma KRT20 concentration at various time points pre- and post aHSCT: a focused analysis comparing completely unaffected (No aGVHD) and severely affected (Cut + GI aGVHD) aHSCT patients (*t*-test: * *p* < 0.05).

Considering this, first we focused our analysis on these two patient cohorts only. Interestingly, the difference in plasma levels of KRT20 between these two patient groups reached significance on day 28 post aHSCT (Figure 5). Notably, day 28 post-aHSCT occurred 27.8 ± 11.4 (mean ± SEM) days before the diagnosis of severe aGvHD in the affected patients, raising the question if KRT20 may have prognostic value. In this venue, it must be noted that average KRT20 levels were at all times lower in patients that belong to the affected group, than in their unaffected controls (Figure 5), and that the difference observed on day 14 post aHSCT (Figure 5) was also very close to reach significance (*p* = 0.0503). Next, we made an attempt to find similar differences between other patient groups, as well (i.e., to test if KRT20 could discriminate between unaffected patients and patients affected by either organ manifestation alone). No such differences have been observed (not shown).

Finally, we performed multiple logistic regression to assess the risk represented by low KRT20 compared to other factors such as age, gender, HLA match, donor-recipient relationship, conditioning regimen, or the blood levels of REG3A and PI3 as controls. In line with others’ findings, we found that age (*p* = 0.0378, OR = 1.324) or day 28 REG3A levels (*p* = 0.0373, OR = 27.89) were risk factors predisposing to the development of gastrointestinal aGvHD. However, at least in our data set, no such link could be demonstrated between plasma PI3 levels and the risk of cutaneous aGvHD, or plasma KRT20 levels and the risk of multi-organ involvement.

Follow-up analysis of the dynamics of KRT20 level before and after aHSCT in patients developing no GvHD compared to patients affected by cutaneous and GI aGvHD (*t*-test/Mann–Whitney test, *p*-values indicated, stars indicate significance).

## 4. Discussion

By analyzing plasma samples obtained from patients undergoing allogeneic hematopoietic stem cell transplantation, this study found considerable individual variability in the levels of the blood protein cytokeratin 20 (KRT20). Our observations indicate that the concentration of circulating KRT20 may be affected by the development of acute graft versus host disease (which may follow aHSCT). Using a discovery and validation design, we provide convincing evidence that, at the time of aGvHD diagnosis, patients diagnosed with either skin- or gut aGvHD present with lower KRT20 levels. These phenomena seem to develop independently from each other, leading to profoundly decreased levels of KRT20 in aGvHD patients with multiple organ involvement (i.e., in individuals affected in the skin and the gut). Unsurprisingly, we showed that patients with grade 2+ moderate to severe aGvHD display the lowest KRT20 levels, suggesting a link between disease severity and the availability of KRT20 in the blood as well.

In addition to the above, we provide circumstantial evidence for the existence of KRT20 dysregulation several days before the diagnosis of aGvHD. In fact, our data raise the question concerning whether this phenomenon represents a pre-existing condition affecting patients weeks before aGvHD (possibly even at the time of transplantation). Clearly, this segment of the study is less compelling, and it is difficult to draw conclusions in this regard for two reasons. First, the patient population most affected (i.e., aGvHD patients with multiple target organ involvement) represent a small fraction of aHSCT patients, and hence our analysis was underpowered. Second, in this particular patient group, KRT20 levels are the lowest and are often barely detectable or undetectable by currently available ELISA assays. This results in a skewed distribution of the data, with data clumping clearly visible at the detection limit. Taken together, a fair and more accurate assessment of the prognostic value of KRT20 would require the recruitment of significantly larger patient cohorts and novel, more sensitive KRT20 assays.

To the best of our knowledge, cytokeratin 20 has not been analyzed in the context of aGvHD or aHSCT before. There have been no studies reported in preclinical models demonstrating that reduced plasma KRT20 maybe predictive of aGvHD, and this is the first study showing that KRT20 may be of clinical value in human aGvHD.

KRT20 is an intracellular cytoskeletal protein, a type I, acidic, simple cytokeratin building antiparallel heterodimers with other members of the broader cytokeratin family (predominantly KRT8) [30]. These, in turn, are oriented into staggered tetramers, finally making up the keratin protofilaments. Intermediate filaments containing KRT20-KRT8 dimers may constitute a considerable, even if relatively small, part of the 10 nm filaments of epithelial cells. Gene expression of KRT20 is largely tissue restricted. KRT20-positive epithelial cells are present mostly in the GI tract (most typically in the esophagus, the stomach, and the small and large intestines). Nevertheless, KRT20 is not strictly gut-restricted, as it is strongly expressed by the urothelia and Merkel cells of the skin, too. Of note, KRT20 proteins, possibly leaking from the epithelia into the blood stream rather than actively secreted from epithelial cells, are readily detectable in the circulation.

In clinical terms, KRT20 is a well-established biomarker of various epithelial neoplasms, as it can be exploited for diagnosis, staging, and prognosis [30,31]. It may reveal the localization of the primary tumor, and it may indicate the extent of chromosomal instability [32,33]. In addition to its characteristic or aberrant expression in some neoplasms, several lines of evidence suggest that tissue expression of KRT20 may undergo changes in non-transformed epithelia exposed to environmental stressors as well. Among others, dysregulation of KRT20 expression has been reported in the stomach induced by H. pylori infection [34], in Barrett’s esophagus related to gastric reflux [35], and in murine DSS-induced colitis [36], as well. Interestingly though, consequences of the perturbation of KRT20 expression remain often elusive. There is evidence that both forced expression, and targeted mutation of KRT20, may be surprisingly well-tolerated by gut epithelial cells, the GI-tract, and the host, even if obvious structural rearrangement of the affected filaments occur [29].

Considering the above, proper functional interpretation of our observations represents a considerable challenge. Possibly the most straightforward explanation for these data may be that low plasma levels of KRT20 are a result of: (a) of decreased production of KRT20 in the gut barrier; (b) decreased leakage of KRT20 from the gut epithelia; and (c) either proteolytic lysis or shortened half-life of soluble KRT20 in the lamina propria, the circulation, or any combination thereof. In our opinion, considering the literature available on KRT20 expression and distribution in the small and large intestine (i.e., the fact that KRT20 is most prominently expressed by well-differentiated epithelial cells protruding in the luminal space [28,29,31]), the events observed in this study may be consequences of external or internal factors affecting differentiated gut epithelial barrier of aHSCT patients developing aGvHD.

As proper function of luminal epithelial cells is a prerequisite of the maintenance of an intact barrier, KRT20 is linked to the barrier, and aGvHD thrives on a disrupted barrier, a plausible explanation may be that patients developing aGvHD, or even patients at risk of developing aGvHD, may be influenced by conditions existing at the time of diagnosis, or maybe even earlier (e.g., possibly at transplantation), thereby affecting KRT20. In other words, we believe that pre-existing stressors of the barrier, being well-documented risk factors of aGvHD as well (such as alterations in the gut microbiota, tissue damage caused by the conditioning regimen, and genetic factors predisposing to aGvHD) may, as a mere side effect, also alter, either directly or indirectly, the production, release, or stability of KRT20 proteins in or around the gut epithelia (their prime target, which is ultimately reflected by lowered KRT20 levels in the blood). In short, our data raise the question concerning whether plasma KRT20 could serve as a marker of gastrointestinal barrier fitness in aHSCT with low KRT20 indicating barrier stress. Certainly, no matter how plausible this hypothesis may sound, hypothesizing the occurrence of tissue-level protein changes based on plasma protein assays alone is a dubious undertaking. The viability of such a “liquid biopsy” approach (i.e., an assessment of gut damage based on plasma protein analysis) has been convincingly demonstrated by the MAGIC algorithm and the example of REG3A, ST2, and TNFR1 in aGvHD [16]. Of note, earlier it has been demonstrated that decreased pre-transplant plasma levels of citrulline, analysed by ELISA, were also associated with increased risk of aGvHD upon aHSCT [24]. As citrulline is a plasma biomarker directly reflecting the mass of functional gut epithelial cells [37,38], these data seem to corroborate the idea that plasma biomarkers of enterocyte fitness may be used in GvHD risk assessment after aHSCT. Nevertheless, it remains to be verified whether KRT20 could be exploited in a similar manner. Hence, further studies are required to clarify the viability of this hypothesis by measuring KRT20 production and tissue distribution in situ both in and around epithelial cells. If dysregulation of KRT20 production, abnormal localization, or a shorter half-life could be convincingly demonstrated in tissue biopsies at or before diagnosis, the stress factors(s) contributing to the changes observed in circulating KRT20 levels may be identified with a reasonable chance as well.

Discovery of novel diagnostic, prognostic, and predictive aGvHD markers is important from both clinical and economic perspective. The costs of aHSCT [39,40], the estimated cost per QALY gained [41,42], the high morbidity and mortality associated with aGvHD, the challenge of treatment resistance [1], and the solid evidence available that development of aGvHD (especially that of severe, grade 3-4 form of the disease) multiplies treatment costs, prolongs hospital stays [40], and may have devastating long-term consequences for survivors seem to support this notion. There is growing demand for robust biomarkers for risk assessment and early diagnosis and for markers to guide the choice of treatment in steroid-refractory aGvHD. Ultimately, such markers have the potential to improve the patients’ quality of life and increase cost-effectiveness of aHSCT, as well. We believe that similar to REG3A, novel markers such as FABP2, citrulline, and probably KRT20 (i.e., plasma markers related to gut barrier immunity, fitness, enterocyte mass and turnover) will contribute to early diagnosis and better risk assessment in aGvHD as they react to the earliest changes in the gut barrier during the development of the disease.

Finally, we believe that the case of KRT20 and citrulline highlights key differences in the applicability of the two most frequently applied study approaches in GvHD biomarker discovery. In recent years, hypothesis-free, mass spectrometry-based proteome profiling has been used with great success for marker identification in aGvHD. This approach has provided unprecedented insight into the dynamics of thousands of serum, plasma, and urine proteins potentially affected by the disease [17,19,43,44,45,46]. On the other hand, this methodology is difficult to standardize, is less suitable for routine clinical use, and it may have difficulties detecting rare events, such as tissue leakage markers or cytokines in the plasma [47]. In contrast, hypothesis-driven studies, mostly relying on ELISA, are limited to the analysis of a few markers and are more prone to specificity-related issues. Nevertheless, ELISA requires less optimization to encompass the full dynamic range of a given marker, detects even rare plasma proteins in a reliable manner, and is easier to standardize and transfer to clinical use. KRT20 and citrulline represent rare, tissue-leakage plasma markers, detected in the low ng-pg/mL range following aHSCT. Such rare markers, particularly citrulline (being a single amino acid), are suboptimal targets for conventional mass spectrometry [48]. However, as these studies demonstrate, they can be linked to GvHD by an ELISA-based, focused study. Taken together, this study supports the notion that different analytical approaches may complement each other in the discovery and validation of clinically relevant plasma biomarkers of aGvHD.

## Figures and Tables

**Table 1:** Discovery set, summary of patient data. Summarized patient data describing the validation set according to age, gender, conditioning regimen, indication and outcome of aHSCT, donor-recipient relationship, HLA match, aGvHD developed, type and extent of organ involvement, disease severity (grade). List of abbreviations: ^1^ALL, acute lymphoblastic leukemia; ^2^AML, acute myeloid leukemia; ^3^CML, chronic myelogenous leukemia; ^4^Haplo, haploidentical donor; ^5^HD, Hodgkin’s disease; ^6^MDS, myelodysplastic syndrome; ^7^MUD, matched unrelated donor; ^8^MM, multiple myeloma; ^9^MPN, myeloproliferative neoplasm; ^10^NHL, non-Hodgkin’s lymphoma.

		No aGvHD	Cutaneous aGvHD	Gastrointestinal aGvHD	Cutaneous and Gastrointestinal aGvHD
Group size (*n*)		10	10	10	9
Age; median (range)		44 (22–58)	41 (20–68)	33.5 (19–57)	58 (21–66)
Gender	Female	3	3	1	4
	Male	7	7	9	5
Conditioning regimen	Myeloablative	6	6	7	2
	Reduced intensity	4	4	3	7
Indication of aHSCT	^1^ALL	2	2	4	1
	^2^AML	4	3	4	2
	^3^CML	0	1	0	0
	^5^HD	1	0	0	0
	^9^MPN	1	1	1	1
	^6^MDS	0	1	1	5
	^8^MM	1	1	0	0
	^10^NHL	1	1	0	0
Donor type	Sibling	3	6	4	2
	^4^Haplo	0	1	1	2
	^7^MUD	7	3	5	5
Status (aHSCT + day 100)	Dead	3	2	2	7
	Alive	7	8	7	2
	Unknown	0	0	1	0
aGvHD grade	0	10	0	0	0
	I	0	4	2	0
	II	0	4	4	4
	III	0	0	1	1
	III-IV	0	0	2	3
	IV	0	2	1	1

**Table 2:** Validation set, summary of patient data. Summarized patient data describing the validation set according to age, gender, conditioning regimen, indication and outcome of aHSCT, donor-recipient relationship, HLA match, aGvHD developed, type and extent of organ involvement, disease severity (grade). List of abbreviations: ^1^ALL, acute lymphoblastic leukemia; ^2^AML, acute myeloid leukemia; ^2^AML/^6^MDS, acute myeloid leukemia/myelodysplastic syndrome; ^3^B-ALL, acute B lymphoblastic leukemia; ^4^B-TH, beta thalassemia; ^5^CR, complete remission; ^6^Haplo, haploidentical donor; ^7^HLH, hemophagocytic lymphohistiocytosis; ^8^MDS, myelodysplastic syndrome; ^9^MDS RAEB-II, myelodysplastic syndrome—refractory anemia with excess blasts; ^10^MF, myelofibrosis; ^11^MM, multiple myeloma; ^12^MPN, myeloproliferative neoplasm; ^13^MUD, matched unrelated donor; ^14^NHL, non-Hodgkin’s lymphoma; ^15^NR, no response; ^16^PreB-ALL, pre acute B lymphoblastic leukemia; ^17^PNH, paroxysmal nocturnal hemoglobinuria; ^18^SAA, severe aplastic anemia; ^19^T-ALL, T-Cell acute lymphoblastic leukemia; ^20^VGPR, very good partial response.

		No aGvHD	Cutaneous aGvHD	Gastrointestinal aGvHD	Cutaneous and Gastrointestinal aGvHD
Group size (*n*)		40	14	7	6
Age; median (range)		43 (19–66)	43 (21–63)	39 (20–46)	36 (19–66)
Gender	Female	19	8	4	5
	Male	21	6	3	1
Conditioning regimen	Myeloablative	28	10	5	4
	Reduced intensity	12	4	2	2
Indication of aHSCT	^1^ALL	0	1	0	0
	^2^AML	17	2	1	1
	^2^AML/^6^MDS	3	0	0	0
	^3^B-ALL	3	1	3	2
	^4^B-TH	1	0	0	0
	^7^HLH	0	0	0	1
	^8^MDS	4	2	0	1
	^9^MDS RAEB-II	0	1	0	0
	^10^MF	4	1	0	0
	^11^MM	0	0	1	1
	^12^MPN	1	0	0	0
	^14^NHL	0	2	0	0
	^16^PreB-ALL	0	1	0	0
	^17^PNH	1	1	0	0
	kidney amyloidosis	1	0	0	0
	Sézary disease	1	0	0	0
	^18^SAA	2	1	1	0
	^19^T-ALL	2	1	1	0
Donor type	Sibling	14	4	3	2
	^6^Haplo	7	3	0	1
	^13^MUD	19	7	4	3
Status (aHSCT + day 100)	Dead	10	3	3	2
	Alive	30	11	4	4
	Unknown	0	0	0	0
aGvHD grade	0	40	0	0	0
	I	0	5	0	0
	II	0	9	4	5
	II-III	0	0	1	1
	III	0	0	2	0
	IV	0	0	0	0

## Data Availability

Not applicable.

## Supplementary Materials

The following supporting information can be downloaded at: https://www.mdpi.com/article/10.3390/biomedicines10030519/s1, Table S1: Discovery data set, detailed patient data and clinical characteristics; Table S2: Validation data set, detailed patient data and clinical characteristics.
